# Neoadjuvant immunotherapy leads to complete pathologic response in locally advanced colon cancer

**DOI:** 10.1002/ccr3.9218

**Published:** 2024-08-06

**Authors:** Lyndsey Sandow, Liana Tsikitis, Charles D. Lopez, Brian Brinkerhoff, Adel Kardosh, Guillaume Pegna, Emerson Y. Chen

**Affiliations:** ^1^ Department of Medicine Oregon Health and Science University Portland Oregon USA; ^2^ Division of Gastrointestinal and General Surgery, Department of General Surgery Oregon Health and Science University Portland Oregon USA; ^3^ Division of Hematology and Medical Oncology Oregon Health and Science University, Knight Cancer Institute Portland Oregon USA; ^4^ Department of Pathology Oregon Health and Science University Portland Oregon USA

**Keywords:** colon cancer, complete pathologic response, immunotherapy, pembrolizumab

## Abstract

Immunotherapy is considered first line in patients with dMMR metastatic colorectal cancer (CRC). Recent studies have also shown promising results with neoadjuvant immunotherapy in locally advanced CRC. We report a case in which neoadjuvant immunotherapy with pembrolizumab resulted in complete pathologic response at time of resection as well as saved the patient the morbidity associated with a hepatectomy. We also completed a scoping review of the literature which suggests promising tumor responses with treatment in dMMR CRC. Further randomized control trials to determine the magnitude of response and optimal regimen are needed.

## 1 INTRODUCTION

Colorectal cancer (CRC) is the third most common cancer and the second most common cause of cancer death in the United states.^1^ The 5‐year survival rate for CRC continues to increase reflecting improvements in screening, surgical techniques, and advances in therapy, including targeted therapies like immunotherapy.^1^

The decision to add immunotherapy hinges on the genetic makeup of the tumor, specifically the mismatch repair pathway which can arise as either a somatic or germline mutation. This genetic aberration is found in 15% of all CRC patients and in sporadic colon cancer is usually caused by methylation of the MLH1 gene promoter.^2^ Other implicated mutations include PMS2, HSM2, MSH6, MLH3, MSH3, and PMS1.^3^

Standard of treatment for CRC cancer varies depending on the stage and MMR status. In locally advanced cases in which adjacent structures are involved or at risk, neoadjuvant therapy with FOLFOX (5‐FU, oxaliplatin, and leucovorin) or CAPEOX (capecitabine and oxaliplatin) can be considered; however, chemotherapy is cytotoxic and associated with significant adverse reactions and morbidity.^4^ In dMMR metastatic cases, however, checkpoint inhibitor immunotherapy is preferred for neoadjuvant therapy and first‐line treatment.^4^

Current evidence suggests that dMMR CRC is less responsive to standard chemotherapy.^5^ Immune checkpoint blockade has shown to be highly effective for patients with metastatic dMMR CRC in first‐line and refractory treatment setting.^6^, ^7^, ^8^, ^9^ Given the significant morbidity associated with chemotherapy and surgery, interest in both better tolerated systemic therapy and non‐operative management is increasing. Success of PD‐1 blockade in the metastatic setting has led to a growing interest of its use in the neoadjuvant setting. A recent study looked at PD‐1 blockade in dMMR locally advanced rectal cancer and showed 100% complete clinical response.^10^ In NICHE, neoadjuvant PD‐1 plus CTLA‐4 was used in dMMR early stage CRC resulting in 100% pathologic response.^11^ These studies suggest complete response without the morbidity associated with traditional chemotherapy and surgery. We report a case in which neoadjuvant pembrolizumab led to a complete pathologic response in stage III CRC and complete a review of current literature on the use of neoadjuvant immunotherapy in resectable CRC.

## 2 CASE HISTORY/EXAMINATION

A 65‐year‐old woman with a past medical history significant for obesity, psoriasis, obstructive sleep apnea, and type 2 diabetes complicated by peripheral polyneuropathy was referred by her primary care physician following a screening colonoscopy notable for a right ascending colonic mass.

## 3 METHODS (DIFFERENTIAL DIAGNOSIS, INVESTIGATIONS, AND TREATMENT)

A CT chest, abdomen, and pelvis was notable a circumferential soft tissue mass involving the cecum, and ascending colon and spanning approximately 8 cm in length. The mass was noted to be contiguous to the right hepatic lobe and about six mildly enlarged lymph nodes in the pericecal region and along the anterior thecal vessels were noted. The patient's carcinoembryonic antigen (CEA) was elevated to 7.8 ng/mL at this time (reference range <3.0 ng/mL). The biopsy of the mass from the colonoscopy was notable for colorectal adenocarcinoma and next generation sequencing showed positive microsatellite instability status, a high tumor mutational burden (24 mutations/Mb), and BRAF V600E, and loss of MSH6 mutations (see Data S1 for other mutations). Immunostains were notable for loss of nuclear expression of MLH1 and PMS2 and MLH1 hypermethylation. The patient was determined to have clinical stage IIIc (cT4b, cN2, cM0) colorectal adenocarcinoma. Given the concern for invasion into the liver capsule requiring liver resection in addition to right hemicolectomy, the patient was offered neoadjuvant treatment.

## 4 CONCLUSION AND RESULTS

**FIGURE 1:**
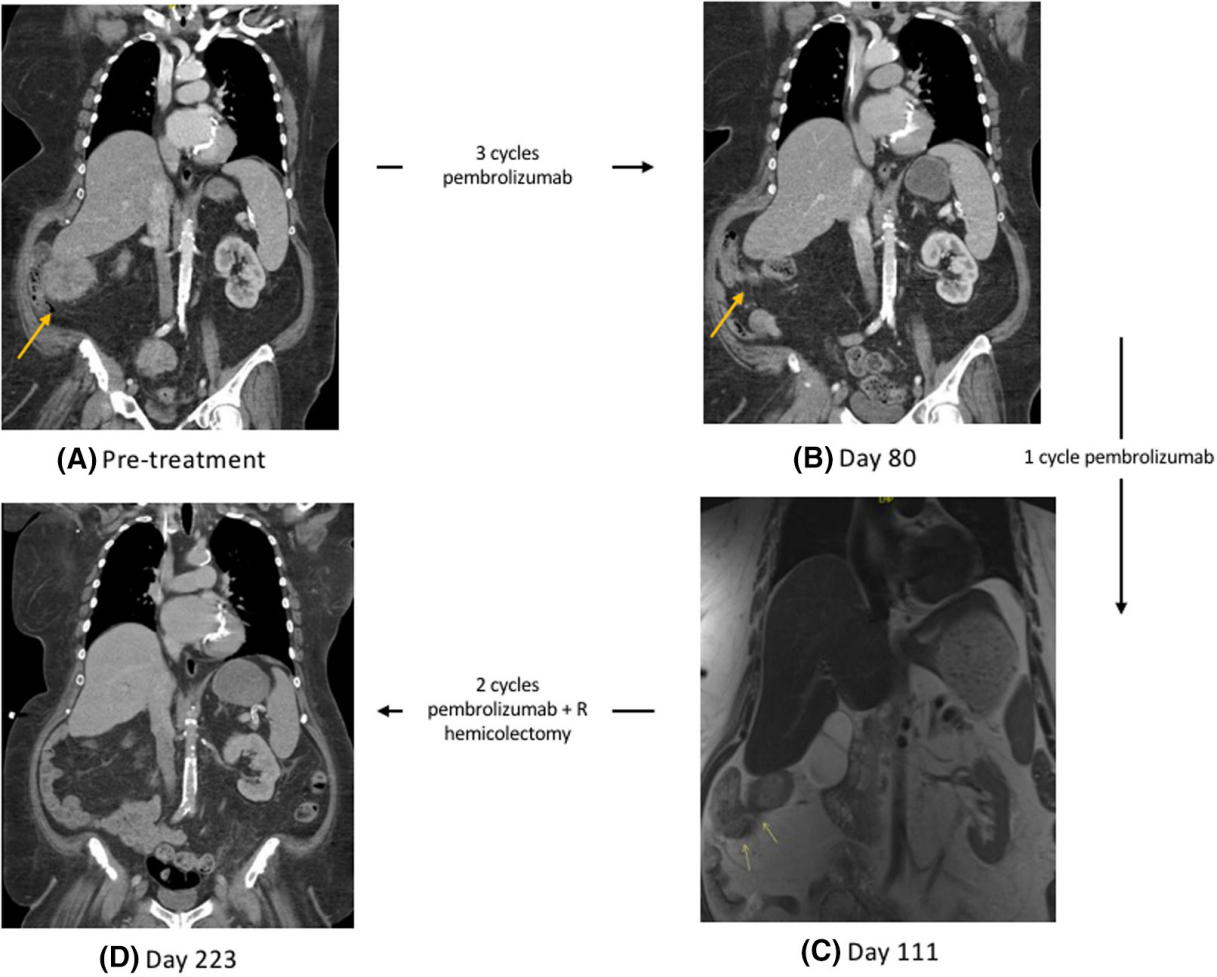
Baseline contrast enhanced CT showing circumferential soft tissue mass contiguous to the right liver inferior margin with question tumor invasion to the liver capsule (A). Follow up contrast enhanced CT following 3 cycles of pembrolizumab and follow up MRI following another 3 cycles of pembrolizumab showing interval decrease in cecal mass without definite involvement with the inferior right hepatic lobe (B, C). MRI post hemicolectomy and pembrolizumab showing post‐surgical hemicolectomy (D).

**FIGURE 2:**
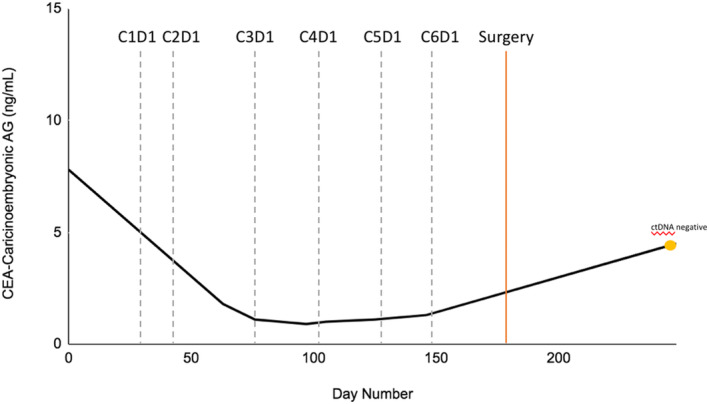
Carcinoembryonic antigen (CEA) trend overtime during neoadjuvant pembrolizumab treatment. Gray dotted line indicates pembrolizumab treatment cycles. Orange solid line indicates time of R hemicolectomy. Yellow point marks when ctDNA was collected and found to be negative.

Following a multidisciplinary discussion, based on patients MSI high status and significant peripheral neuropathy limiting treatment with FOLFOX, patient was started on neoadjuvant pembrolizumab 200 mg every 3 weeks. After three cycles of pembrolizumab, a repeat CT chest, abdomen, and pelvis showed interval decrease of the cecal mass without involvement of the liver capsule and similar size and appearance of the mildly enlarged lymph nodes along the right colic vessels without evidence of new adenopathy or metastatic disease (Figure 1). After the fourth cycle, an MRI abdomen was completed and notable for lack of overt involvement of the inferior right hepatic lobe with the cecal mass, continued decrease in size of the cecal mass, and remonstrated adenopathy. The patient's CEA was also followed and decreased to 1.0 ng/mL during this time (Figure 2).

**FIGURE 3:**
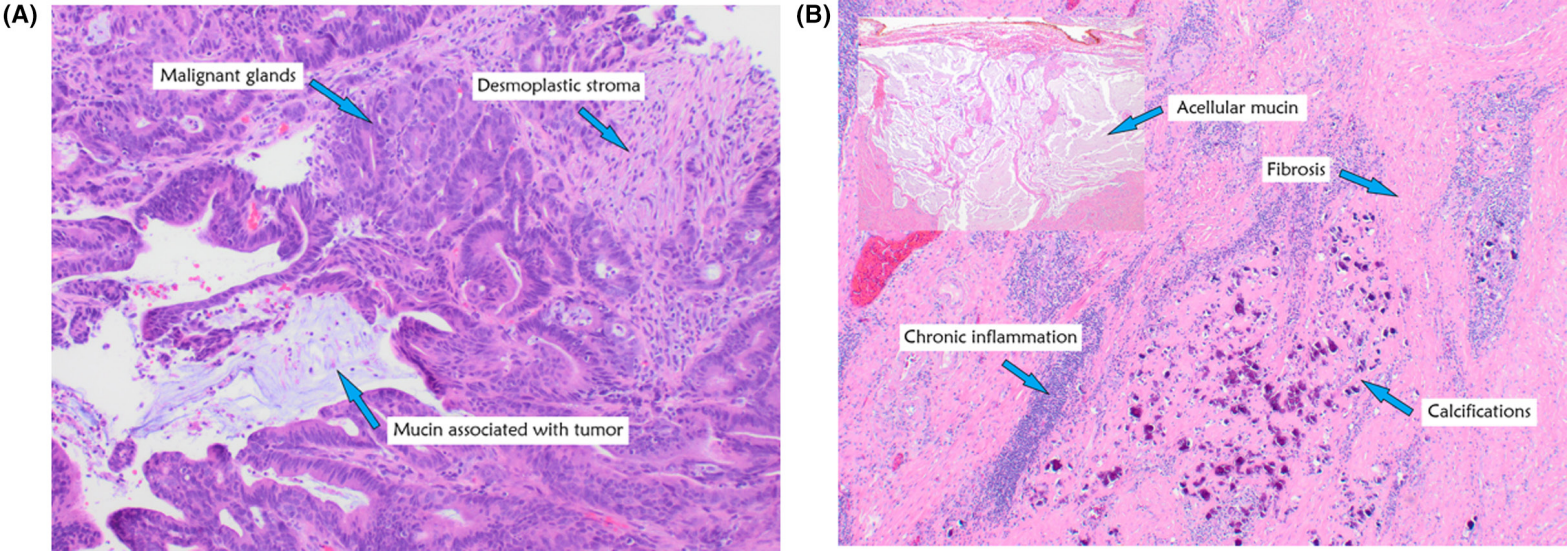
(A) Pre‐treatment biopsy from the ascending colon mass (high‐power). Extensive malignant glands with luminal mucin and acute inflammatory cells are present. There is associated desmoplastic stroma, suggesting invasion at least into the submucosa. A pathologic diagnosis of “Invasive adenocarcinoma, moderately‐differentiated” was rendered. (B) Post‐treatment resection from the right hemicolectomy specimen (low‐power). A 3.0 cm tumor bed in the right colon was identified and extensively sampled histologically. Representative images demonstrate treatment‐related changes, including chronic inflammation, fibrosis, dystrophic calcifications, and subserosal acellular mucin. No residual carcinoma is present, consistent with complete treatment response (Score 0, College of American Pathology synoptic report) (Score 1, Ryan Classification).

The patient completed three more cycles (a total of six cycles) prior to a right hemicolectomy. During the procedure, there was no evidence of residual disease or liver involvement and the patient was spared a hepatectomy. Pathology was notable for no residual invasive carcinoma, negative margins, and a lack of lymphovascular or perineural invasion. Twenty lymph nodes were sampled and all were negative for malignancy (Figure 3). There were no post‐operative complications from immunotherapy. The surgical pathology collected at this time showed a complete pathologic response without any residual invasive carcinoma present. At present, patient is currently undergoing routine surveillance with most recent imaging without evidence of disease. Patient's treatment course was complicated by a right distal deep venous thrombus treated with apixaban, psoriasis, and inflammatory arthritis requiring topical and low‐dose prednisone, and enteritis that resolved with prednisone and budesonide, as well as diarrhea requiring steroids which lead to a 1‐week delay in cycle 4, inflammatory arthritis requiring oral steroids and psoriasis flares requiring topical steroids. CtDNA testing was also completed and negative post hemicolectomy.

## 5 DISCUSSION

In gastrointestinal cancers, treatment with immunotherapy is relatively new with the first FDA approval for the use of pembrolizumab in patients with dMMR CRC after prior treatment in May 2017.^7^ Following KEYNOTE‐177, immunotherapy became first‐line treatment in dMMR metastatic CRC patients after improved progression free survival compared to chemotherapy (median 16.5 vs. 8.2 months).^6^ Despite these promising results, there are few studies evaluating neoadjuvant immunotherapy for non‐metastatic CRC. In this paper, we present a case in which neoadjuvant pembrolizumab was used in stage 3 colon cancer leading to a complete pathologic response at time of resection. We also completed a scoping review of high evidence‐based studies evaluating the use of neoadjuvant immunotherapy in resectable CRC.

In our review, a total of three meta‐analysis, three systematic reviews, seven clinical trials, and one randomized control trials met inclusion criteria (Figure 1; Table 1) (Data S1). Six of the trials had a primary outcome of pathologic complete response (pCR). Of these studies, the end point was met in 27%–100% of cases.^11^, ^14^, ^15^, ^16^, ^17^, ^18^ This wide variation in response rates is likely due to the heterogeneity of MMR status among the studies. For example, in Chalabi et al study, a 100% (20/20) pCR was noted in the dMMR subset while the pMMR subset had pCR of 48% (13/27).^11^ As expected, the lower pCR rates were noted in studies which included pMMR patients or reported results based on MMR subgroup analysis suggesting MMR status as a driving factor.

**TABLE 1:** Trial characteristics of included meta‐analysis and clinical trials.

Trial name	Number of patients included	% MSI‐high (dMMR)	Outcomes assessed	Treatment	Pathologic complete response
Chalabi et al.	40	51%	Pathologic response	Ipilimumab + nivolumab	pMMR 4/15 (27% [95% CI [8–55]]) dMMR 20/20 (100% 95% CI [86–100])
Lin et al.	30	3%	Pathologic complete response	SCRT + CAPOX + Camrelizumab	pMMR 13/27 (48.1%) dMMR 1/1 (100%)
Li et al.	1503^a^	NR	Objective response rate	Anti‐PD1/PD‐L1	pMMR 1/1 (100% [CI NR]) dMMR 13/27 (48.1% [CI NR])
Hu et al.	53	100%	Pathologic complete response	Tropalimab + celecoxib	15/17 (88% [95% CI 64–99])
Zhou et al.	410^a^	40%	Pathologic complete response	Neoadjuvant immunotherapy	167/373 (44.6% [95% CI 36–62])
Ludford et al.	35	100%	Pathologic complete response	Anti‐PD1/PD‐L1	67% [95% CI 38–88]
Rahma et al.	185	NR	NAR score	FOLFOX + CRT + pembrolizumab	31.90%
Yoshino et al.	37	100%	Pathologic complete response	Nivolumab + CRT	11/37 (30% [90% CI 18–44])
Jin et al.	15 articles^a^	NR	Objective response rate	Anti‐PD1/PD‐L1 or anti‐CTLA4	11% [95% CI 8–15]
Overman et al.	74	100%	Objective response rate	Nivolumab	3% [95% CI NR]
Sun et al.	60	100%	Total clinical effective rate	FOLFOX + SHR 1210	NR

^a^ Meta‐analysis.

While all studies evaluated either anti‐PDL1/PD1 or anti‐CTLA4 drugs, some tested these drugs in combination with either chemotherapy (FOLFOX or CAPOX), or chemoradiotherapy, or radiation. One meta‐analysis and two clinical trials assessed objective response rate as their primary outcome. In these studies, the primary endpoint was met and found to be 33% and 23%.^8^, ^12^, ^13^ Finally, one study used the NAR score as the primary endpoint and was found to be not statistically significant compared to control.^19^ This trial was the only trial/meta‐analysis which did not have a conclusion supporting the use of neoadjuvant immunotherapy.^19^ Interestingly, the pCR (31.9%) was similar to other trials; however, the NAR score which is a surrogate for overall survival was not statistically significant.^19^ Regardless, review of current high evidence based trials suggests that neoadjuvant immunotherapy in resectable colon cancer leads to overall improved tumor response in dMMR tumors and mixed response in pMMR tumors.

Of the included studies, PD‐1 inhibitors were evaluated the most. All of the clinical trials included in the review included a PD‐1 inhibitor in the intervention arm. The most frequently used PD‐1 inhibitor was nivolumab (*n* = 3).^8^, ^11^, ^16^ Other PD‐1 inhibitors used included toripalimab, camrelizumab, and pembrolizumab.^15^, ^18^, ^19^, ^20^ In Chalabi et al. ipilimumab and nivolumab were used together.^11^ Based on these limited studies, single agent PD1/PDL1 was not inferior to combined checkpoint inhibition or in combination with chemotherapy.^8^, ^12^, ^18^, ^21^ While Chalabi et al. did show 100% pCR in dMMR patients, it is possible that this response is driven by MMR status.^11^ There are many ongoing trials aimed at determining the role of neoadjuvant therapy in resectable CRC and preferred regimen (Table 2).

**TABLE 2:** Current clinical trials evaluating neoadjuvant immunotherapy in locally advanced or resectable colorectal cancer.

Study title	Identifier	Country	Phase	Study size	Intervention	Primary endpoint	MMR status
Envafolimab as neoadjuvant immunotherapy in resectable local advanced dMMR/MSI‐H colorectal cancer	NCT05371197	China	II	26	Envafolimab	Pathologic complete response	dMMR
Toripalimab with or without celecoxib as neoadjuvant therapy in resectable dMMR/MSI‐H colorectal cancer (PICC)	NCT03926338	China	I/II	69	Toripalimab + celecoxib	1. Rate of treatment related surgery delay. 2. Frequency of 3/4 adverse events	dMMR
Neoadjuvant treatment with mFOLFOXIRI Plus cadonilimab (AK104) versus mFOLFOX6 in locally advanced colorectal cancer (OPTICAL‐2)	NCT05571644	China	II	82	mFOLFOXIRI + cadonilimab	Pathologic complete response	NR
Immunotherapy in locally advanced rectal cancer (AVANA)	NCT03854799	Italy	II	101	Avelumab + capecitabine + radiation	Complete pathologic response	NR
Pembrolizumab for locally advanced, irresectable, non‐metastatic dMMR colorectal cancers (PUMA)	NCT05131919	Netherlands	II	25	Pembrolizumab	Objective response rate	NR
Neoadjuvant pembrolizumab in stratified medicine–colorectal cancer (NEOPRISM‐CRC)	NCT05197322	UK	II	32	Pembrolizumab	Pathologic complete response	dMMR

Based on the review completed, the results of neoadjuvant treatment with immunotherapy in CRC are promising. It seems to lead to greater tumor response, especially in dMMR tumors. Given these promising results, translating the use of neoadjuvant immunotherapy in pMMR tumors with microsatellite instability features conferred by high tumor mutation burden and POLE mutations offers other potential treatment possibilities; however, more research is needed.^21^, ^22^ In addition, it seems to have a favorable overall safety profile when compared to standard chemotherapy. Our patient received a total of six cycles of pembrolizumab prior to resection at which time a complete pathologic response was noted. While our patient was motivated for surgery, based on review neoadjuvant immunotherapy in dMMR colorectal tumors offers high potential for pCR negating the need for surgical resection and sparing patient's the morbidity associated with resection. In addition, the significant response from the immunotherapy saved the patient from a right hepatectomy as the lesion was initially contiguous with the right lobe. Finally, following surgical resection, ctDNA was used to monitor patient's disease status. The negative result spared the patient adjuvant chemotherapy.

With regard to adverse events, our patient had enteritis, inflammatory arthritis, and a psoriasis flare while undergoing immunotherapy, and while her treatment was delayed, she was able to complete six cycles (4.5 months of therapy). Despite this, she still achieved complete pathologic response at the time of resection. The duration of treatment varied depending on trial; however, our case suggests that complete pathologic response can be achieved with shorter course of treatment perhaps further limiting risk for adverse events and morbidity.

Based on the case presented and literature review, neoadjuvant immunotherapy could provide durable tumor response in the neoadjuvant setting in CRC. However, further randomized control trials are needed to confirm the magnitude of clinical benefit, to detail potential surgical complications, and to fine‐tune the specific immunotherapy treatment regimens.

## AUTHOR CONTRIBUTIONS

**Lyndsey Sandow:** Conceptualization; data curation; formal analysis; investigation; writing – original draft; writing – review and editing. **Liana Tsikitis:** Writing – review and editing. **Charles D. Lopez:** Writing – review and editing. **Brian Brinkerhoff:** Visualization; writing – review and editing. **Adel Kardosh:** Writing – review and editing. **Guillaume Pegna:** Writing – review and editing. **Emerson Y. Chen:** Conceptualization; methodology; supervision; writing – review and editing.

## FUNDING INFORMATION

This research received no specific grant from any funding agency in the public, commercial, or not‐for‐profit sectors.

## CONFLICT OF INTEREST STATEMENT

All authors have no conflicts of interest to disclose.

## ETHICS STATEMENT

This material is the authors' own original work, which has not been previously published elsewhere.

## CONSENT

Case report reviewed by Oregon Health and Science University IRB. No PHI was obtained or shared. No identifiable objects were included in the report. The HIPPA waiver and Request for Determination were approved by the OHSU IRB. Written informed consent was obtained from the patient to publish this report in accordance with the journal's patient consent policy.

## Supporting information


Data S1.

Data S2.

## Data Availability

The data supporting this study can be found in the included supplemental material.
