# Supplementary material for: Neoadjuvant immunotherapy leads to complete pathologic response in locally advanced colon cancer
Source: Clin Case Rep. 2024 Aug 6;12(8):e9218. doi: 10.1002/ccr3.9218 (PMC11303659; doi:10.1002/ccr3.9218)
Supplement: Supplementary file 1 — Data S1. [file CCR3-12-e9218-s001.docx]

**Materials and Methods**

Systematic search of the literature

We conducted a scoping review to characterize and summarize the current research and evidence in neoadjuvant immunotherapy in resectable colon cancer with the primary aim to provide an overview of the current literature. We searched MEDLINE (OVID) up to 24 March 2023. The search strategy was developed and executed by a librarian scientist (A.H.). Medical subject headings (MeSH) terms were identified through a limited review of the literature and agreed upon by the research team. The reference lists of studies identified and included were reviewed to identify other relevant studies.

Eligibility Criteria

We considered studies evaluating neoadjuvant immunotherapy in resectable colorectal cancer. Only systematic reviews, meta-analysis and clinical trials were included. Studies published before 2014 were excluded. Studies which exclusively evaluated late stage, metastatic, or recurrent colorectal cancers were excluded. Studies which exclusively evaluated chemotherapy, radiation, surgery, or any combination of the previous were excluded; studies evaluating immunotherapy were included. Finally, studies aimed at neoadjuvant treatment results were included; studies evaluating biomarkers and imaging modalities as primary endpoints were excluded. There were no exclusion criteria based on patient demographic data, geographic location, or setting. The search was limited by English language.

Article Selection

All articles were screened for title and abstracts to exclude unrelated citations. All citations were reviewed by L.S. For final inclusion of articles, E.C. reviewed articles. When discrepancies were identified, researchers discussed the discrepancies and resolved them. Reviewers were not blinded to journal name or article author.

Data extraction and analysis

A data chart was created in Excel. Extracted information included study characteristics (year of publication, country of origin, study design, and sample size), Intervention details (intervention arms, evaluated therapies, and dosing specifics), and measured outcomes (primary outcomes, safety outcomes). A comments section was used for other relevant information. Missing data was treated as not reported.

**Results**

Literature Search and Selection Process

Through our search, we identified 2,600 unique records (Figure 1). These records were organized by levels of evidence. A total of 177 systematic reviews and 197 randomized control trials/clinical trials were identified. After screening these 374 abstract and titles, 20 systematic reviews and 17 randomized control trials/clinical trials were deemed relevant. Of these studies, 14 were identified as meeting criteria by full text screening. There were 4 additional studies included from references lists. In the end, a total of 14 articles were included in this scoping review.

Study Characteristics

We included 3 systematic reviews, 3 meta-analysis, and 1 randomized control trial, and 7 clinical trials. Characteristics of the included studies are listed in Table 1. All included trials were published after 2015 with the majority of the studies published after 2020 (n=11). The sample size of the studies was between 10 patients and 410 patients. Most of the trials (n= 11) targeted colorectal cancer while 3 studies looked specifically at rectal cancer. One study was tumor agnostic however 27/37 patients enrolled had colorectal cancer. Of the clinical trials included in this scoping review, there were 3 multi center trials and 4 single center trials. Most of the trials were also phase 2 trials (n = 5) and 1 phase 1 trial was reported.

Outcomes Assessed

Two of the meta-analysis reported on objective response rates [12, 13]. The third meta-analysis reported on pathologic complete response [14]. The primary outcomes assessed in the trials was varied however the most common reported primary outcome was some variation of pathologic response including complete pathologic response and major pathologic response (n= 4)[11, 15-18] . Other outcomes assess include the objective response rates, total clinical effective rate, and the neoadjuvant rectal (NAR) score which is a surrogate endpoint in clinical trials for determination overall survival [19, 20].

Adverse Events and Safety Outcomes

The most frequently reported adverse drug reaction were mild events (grade I-II). The trials that included mild adverse events noted between 59% and 49%[8, 15]. The most common grade I-II treatment related adverse events were fatigue and diarrhea [15, 21]. The most commonly reported grade III adverse drug reactions were increased liver enzymes (n=2), increased amylase/lipase (n=1), hypertension (n=2), leukopenia (n=2), anemia (n=4), neutropenia (n=4)and gastrointestinal reactions (n=4) [8, 15, 17, 18, 20, 22].

Treatment Regimens

All treatment regimens included in this study evaluated immunotherapy in the neoadjuvant setting. Three of the clinical trials looked at immunotherapy monotherapy while four of the clinical trials looked at immunotherapy with chemotherapy and/or radiation [11, 15, 16, 20, 21, 23]. The included chemotherapy regimens were FOLFOX6 (leucovorin, fluorouracil, and oxaliplatin) and CAPOX (capecitabine and oxaliplatin) [17, 23]. The most common immunotherapy studied was PD-1 inhibitors (n=7) and included: toripalimab, nivolumab, SHR-121, carmelizumab, and pembrolizumab [8, 11, 15, 16, 18, 20, 23]. One study looked at both ipilimumab and nivolumab [11]. In addition to the above regimens, the meta-analysis also included studies looking at tremelimumab, durvalumab, and avelumab[12, 13].

Summary Conclusions

Across all meta-analysis, the conclusions were consistent. Each suggested that neoadjuvant immunotherapy could lead to improved clinical responses, especially in the dMMR patients [12-14, 18]. Across the clinical trials, similar conclusions were reached with the exception of the Rahma et al study [20]. The favorable trials (n=7) suggested that neoadjuvant immunotherapy was effective treatment. The Rahma et al study did not suggest that the addition of pembrolizumab improved the primary end point of the NAR score [20]. The most common limitations noted in these studies was the small sample size and the need for more longer term follow up data. Among the meta analysis limitations included inconsistent neoadjuvant immunotherapy regimens, limited number of studies/patients, and lack of randomized controlled trials.
