# Supplementary material for: Neoadjuvant immunotherapy leads to complete pathologic response in locally advanced colon cancer
Source: Clin Case Rep. 2024 Aug 6;12(8):e9218. doi: 10.1002/ccr3.9218 (PMC11303659; doi:10.1002/ccr3.9218)
Supplement: Supplementary file 2 — Data S2. [file CCR3-12-e9218-s002.docx]

**Supplement B**

**Alterations of strong clinical significance:**

BRAFp.V600E

MSH6 p.K247fs*32

**Alterations of potential clinical significance:**

ARID1A p.R1276* and p.G277fs*123

ATRX p.K955fs*10 and p.K1764fs*10

FBXW7 p.G666fs*28

PTCH1 p.R1308fs*64

SETD2 p.N1224fs*5

SMARCA2 p.I1452fs*1
